# Diversité des mouches synanthropes et leur potentiel de transmission des maladies diarrhéïques à Maroua (Extrême-Nord, Cameroun)

**DOI:** 10.11604/pamj.2021.38.410.24687

**Published:** 2021-04-29

**Authors:** Daniel Amani Dawaye, Moussa Djaouda, Eric Moïse Bakwo Fils

**Affiliations:** 1 Département des Sciences Biologiques, Faculté des Sciences, Université de Maroua, Maroua, Cameroun,; 2 Département des Sciences de la Vie et de la Terre, Ecole Normale Supérieure, Université de Maroua, Maroua, Cameroun

**Keywords:** Mouches, synanthropie, diarrhée, Maroua, Flies, synanthropy, diarrhea, Maroua

## Abstract

**Introduction:**

les voies de transmission des maladies diarrhéiques font parfois intervenir les mouches synanthropes, comme vecteurs mécaniques des germes pathogènes. Ce travail avait pour objectif d´évaluer la diversité des mouches synanthropes et de déterminer leur implication potentielle dans la transmission des maladies diarrhéiques dans la ville de Maroua.

**Méthodes:**

des séances de captures des mouches ont été effectuées par saison dans 12 quartiers, en cinq points différents et en trois périodes de la journée correspondant aux différents niveaux d´ensoleillement. Plusieurs clés d´identification des diptères et des analyses microbiologiques en laboratoire ont été utilisées pour estimer la biodiversité et le portage des microorganismes par les mouches synanthropes. Les données collectées ont été soumises à des analyses écologiques et statistiques.

**Résultats:**

huit espèces de mouches synanthropes appartenant à quatre familles ont été identifiées dans la ville de Maroua et la répartition de ces espèces a varié en fonction des saisons, des quartiers et de la période de la journée (P<0,05). Musca domestica et Chrysomya putoria ont été les espèces les plus abondantes, dans les quartiers où les activités de transformation agroalimentaire et l´élevage sont intenses notamment Hardé, Pont-vert, Doualaré, Kongola et Makabaye. Escherichia coli a été plus porté que les salmonelles et le portage des bactéries par les mouches synanthropes a été abondance-dépendant.

**Conclusion:**

la diversité des mouches synanthropes varie en fonction des activités anthropiques, de la saison de l´année et de la période de la journée. Ces mouches constituent d´importants vecteurs mécaniques potentiels des bactéries fécales pathogènes à Maroua.

## Abstract (en)

**Introduction:**

synanthropic flies are sometimes involved in the transmission of diarrheal diseases as mechanical vectors of pathogenic bacteria. The purpose of this study was to assess the diversity of synanthropic flies and to determine their potential involvement in the transmission of diarrheal diseases in the city of Maroua.

**Methods:**

fly catching sessions were carried out per season in 12 wards, in five different sites and in three moments of the day, corresponding to the different daily sunshine hours. Multiple keys for the identification of diptera and of microbiological analyzes in the laboratory were used to estimate the biodiversity and the portage of microorganisms by the synanthropic flies. We carried out an ecological and statistical analyses of collected data.

**Results:**

eight species of synanthropic flies belonging to four families were identified in the city of Maroua and the distribution of these species varied according to the seasons, sites and moment of the day (p<0.05). Musca domestica and Chrysomya putoria were the most numerous species detected in the sites where the activities of agri-food processing and livestock were intense, notably Hardé, Pont-vert, Doualaré, Kongola and Makabaye. Escherichia coli was more involved than Salmonella spp.in the transmission of diarrheal diseases and the portage of bacteria by synanthropic flies was abundance-dependent.

**Conclusion:**

the diversity of synanthropic flies varies as a function of anthropogenic activities, season of the year and moment of the day. These flies are important potential mechanical vectors of fecal pathogenic bacteria in Maroua.

## Introduction

Les maladies diarrhéiques constituent un problème majeur de santé publique dans le monde. Environ 1,7 milliard de cas et près de 750 000 décès de diarrhées d´enfants de moins de cinq ans sont enregistrés chaque année dans le monde. Les maladies diarrhéiques sont classées comme étant la deuxième cause de décès d´enfants de moins de cinq ans et constituent un motif fréquent de consultation, surtout dans les pays en voie de développement où l´hygiène fécale et alimentaire est précaire [1]. Au Cameroun, le taux de morbidité due aux maladies diarrhéiques est estimé à 5,57% et le taux de mortalité à 5,01%. À Maroua, le taux de morbidité est de 5,8 % et le taux de mortalité de 4,6% [2]. Les mouches synanthropes jouent un rôle primordial dans l´épidémiologie des maladies diarrhéiques avec des conséquences graves sur la santé humaine. Elles sont fréquemment rapportées comme étant la cause de la contamination par des agents pathogènes humains [3]. Ces mouches se nourrissent de la matière organique en décomposition, contenant de nombreuses espèces de microorganismes souvent pathogènes pour l´Homme. Elles contaminent les aliments de l´Homme par un simple contact lorsqu´elles s´y posent, laissant parfois des germes pathogènes responsables des maladies diarrhéiques. Il a été prouvé que les microorganismes transportés par les mouches peuvent non seulement séjourner au niveau des pièces buccales, mais peuvent aussi s´y multiplier ou traverser le tractus gastro-intestinal pour être rejetés avec les fèces. Les diptères Muscidae notamment du genre Musca ont été reconnus comme hôtes de transport de plus de 100 germes pathogènes de natures différentes (œufs d´helminthes, virus, protozoaires et bactéries) [4]. Les mouches résistent aux toxines sécrétées par les bactéries, notamment celles de *Escherichia coli* entéro-hémorragiques, et sont de ce fait susceptibles de les déposer sur les aliments [5]. Elles transmettent aussi des salmonelles, agents responsables de la fièvre typhoïde et autres salmonelloses chez l´homme [6]. Elles sont de ce fait considérées comme des vecteurs mécaniques et biologiques des microorganismes avec un taux de transmission dépendant du nombre de pathogènes déposés [7,8].

En Afrique, quelques études sur la diversité des mouches synanthropes ou sur leur potentiel de transmission des germes pathogènes ont été menées en Afrique du sud, en Gambie, au Togo, au Nigéria etc., mais jamais en associant les deux aspects. Au Cameroun en général et dans la région de l´Extrême-Nord en particulier, aucune étude n´a été menée ni sur la diversité des mouches synanthropes ni sur leur potentiel de transmission des germes pathogènes. Maroua est l´une des villes les plus fréquemment touchées par l´épidémie de choléra au Cameroun [9]. Dans cette ville, plusieurs facteurs sont susceptibles d´accroître l´incidence des maladies diarrhéiques. Il s´agit notamment de la mauvaise gestion des ordures, des conditions d'insalubrité, de l´augmentation de la population liée à l´exode rural, et des variations climatiques. Les animaux tels que les bovins, les ovins et la volaille sont très souvent rencontrés dans les concessions où leurs excréments attirent les mouches qui y pondent leurs œufs. Les mauvaises conditions d´hygiène et le faible niveau d´assainissement dans la ville sont favorables au développement des mouches synanthropes. Les aliments de l´Homme sont le plus souvent exposés aux mouches aussi bien dans les maisons que dans les marchés. Très peu d´informations sont cependant disponibles sur la diversité et sur l´implication potentielle des mouches synanthropes dans la propagation des maladies diarrhéiques.

L´objectif général de ce travail était de déterminer la biodiversité des mouches synanthropes et leur potentiel de transmission des maladies diarrhéiques dans la ville de Maroua. Plus spécifiquement, il a été question de: 1) déterminer la diversité des mouches synanthropes en fonction des périodes, des localités et des saisons, et les influences des facteurs environnementaux locaux sur l´abondance des mouches synanthropes dans la ville de Maroua; 2) déterminer le potentiel des mouches synanthropes à transmettre les maladies diarrhéiques, et les influences des facteurs environnementaux locaux sur leurs capacités à transporter les germes des maladies diarrhéiques.

## Méthodes

**Description de la zone d´étude**: la ville de Maroua est située entre 10° 29´ et 10° 41´ de latitude Nord et 14° 15´ et 14° 27´ de longitude Est et à une altitude moyenne de 400 m. Le relief de la ville est très peu variable avec moins de 20 m de différence d'altitude entre les points les plus extrêmes [10]. La ville de Maroua a une superficie de 56 km^2^ et une population estimée à 201 371 habitants [11]. Cette localité est soumise à un climat du type soudano-sahélien caractérisé par l´alternance de deux saisons, une saison sèche qui va du mois d´octobre au mois de mai et une saison des pluies allant de juin à septembre [12]. La pluviométrie annuelle est d´environ 800 à 900 mm [13]. Les pluies sont plus régulières et plus abondantes aux mois de juillet et d´août. La température de l´air oscille entre 18°C aux mois de décembre et janvier, et 45°C, aux mois de mars et avril. La moyenne annuelle de ce paramètre est comprise entre 26 et 28°C [14]. La ville de Maroua est traversée par deux mayos, cours d´eau saisonniers tarissant en saison sèche: le mayo Kaliao et le mayo Tsanaga [10]. De plus, cette localité présente un niveau de développement urbain essentiellement marqué de carence en matière d´assainissement qui, expose sa population à un risque élevé de contamination par les maladies liées à l´insalubrité.

**Choix de sites de capture des mouches**: pour une bonne représentativité de la zone d´étude, les quartiers dans lesquels les mouches ont été capturées ont été choisis après une enquête faite sur toute l´étendue de la ville. Les données relatives à l´écologie des mouches telles que la présence des dépotoirs, des abattoirs, le niveau d´assainissement des quartiers, etc. ont été collectées. Ces données ont été soumises à une Analyse des Correspondances Multiples (ACM) suivie d´une Classification Hiérarchique Ascendante (CHA) afin de stratifier les quartiers de la ville de Maroua. Deux quartiers ont été choisis dans chacun des groupes formés par la CHA.

**Détermination de la structure et de la diversité des communautés des mouches synanthropes**: deux campagnes de capture de mouches ont été effectuées par saison, respectivement le 9 et le 28/06/2019 (saison de pluies), et 30/11 et 18/12/2019 (saison sèche). Dans chacun des sites de capture retenus, les échantillonnages ont été effectués en cinq points caractérisant le mieux les quartiers, distants d´au moins 100 m les uns des autres. Pour chaque campagne, les captures de mouches ont été effectuées au même moment dans les 12 quartiers à trois périodes respectives de la journée, 8h 00-9h 00, 12h 00-13h 00 et 16h 00-17h 00, correspondant à des niveaux d´ensoleillements différents. Ce découpage a été fait en raison de la variation des activités des mouches au cours de la journée. Les mouches adultes des deux sexes ont été collectées grâce à des pièges de Bawa *et al*. [8] modifiés. Les pièges étaient constitués de bouteilles en polyéthylène téréphtalate de 1,5 L, d´une eau minérale vendue localement, lavées à l´eau distillée, puis séchées. Les extrémités antérieures des bouteilles ont été coupées à une hauteur de 11 cm, de telle façon que l´on peut superposer les deux parties en renversant le bouchon vers l´intérieur de la boîte. L´appât, un morceau de 40g de boyaux de bœuf fermentés pendant 12 heures, a été déposé au fond de chaque piège ainsi constitué pour attirer les mouches. Le temps d´exposition de l´appât était de 5 min. En effet, après plusieurs essais, il a été constaté qu´après les 5 min, la diversité biologique des mouches attirées par l´appât n´augmente plus. Après ce temps d´exposition du piège contenant l´appât, la partie supérieure coupée de la bouteille a été rapidement déposée sur le piège et toutes les mouches attirées par l´odeur de l´appât ont été enfermées. L´ensemble du kit a été condamné à l´aide du scotch et a été mis au frais. Toutes les mouches échantillonnées ont été endormies, puis isolées de l´appât et collectées dans des sacs en plastique. L´identification des spécimens de mouches capturées a été faite à l´aide des clés d´identifications usuelles [15-21].

**Evaluation du potentiel de transmission des maladies diarrhéiques par les mouches**: les mouches des diverses espèces ont été capturées dans les mêmes endroits et aux mêmes périodes précédemment présentés pour évaluer le potentiel de transmission des maladies diarrhéiques par ces organismes. Des petits bocaux à couvercle préalablement lavés à l´eau bouillante et rincés à l´alcool à 90° ont été utilisés pour collecter les mouches. Les mouches fauchées ont été mises dans des sacs en plastique stériles dont les étiquettes portaient la saison, le quartier et la période de capture. Les mouches ont été ensuite transportées au laboratoire et mises au frais afin de les immobiliser. Les coliformes et *Escherichia coli*, bactéries indicatrices de contamination fécales et *Salmonella sp*. ont été recherchés sur le corps des mouches, isolés, identifiés et dénombrés.

**Isolement des coliformes, *Escherichia coli* et salmonelles**: après identification des spécimens, cinq mouches de chacune des espèces par quartier ont été introduites dans des tubes à essai contenant 10 mL d´eau physiologique stérile. Puis, les tubes contenant les mouches ont été vortexées à 1000 trs/min pendant 5 secondes dans le but de détacher le maximum des bactéries portées par ces mouches [4]. Un volume de 100 mL du contenu de chaque tube a été ensemencé sur gélose Hektoen selon la technique de platage. La gélose ainsi ensemencée a été incubée à 37°C pendant 24 heures. Les colonies présomptives des coliformes, salmonelles et *Escherichia coli* ont été dénombrées et soumises aux tests d´identification pour confirmation. Le nombre de bactéries par mouche du groupe k (nk) a été calculé selon la formule:

$$n_{k}=\frac{n_{i}v_{x}}{v_{i}x}$$

où n_i_: abondance des bactéries dans le volume v_i_d´eau physiologique ensemencée sur gélose Hektoen; v_x_: volume d´eau physiologique, contenant x mouches, agité pour décrocher les bactéries.

Au bout de l´incubation à 37°C pendant 18 à 24h, les caractères culturaux des colonies bactériennes sur gélose Hektoen ont été utilisés pour identifier des colonies présomptives. Ainsi, toute colonie bactérienne jaune, plate à centre bombé avec un contour régulier et de taille comprise entre 2-3 mm de diamètre a été considérée comme présomptive de *Escherichia coli*. Toute colonie verte ou verte à centre noir a été considérée comme présomptive de Salmonelles. Les colonies jaunes ne présentant pas les caractères de *Escherichia coli* ont été considérées comme présomptives des coliformes. Les colonies suspectes de coliformes, *Escherichia coli* et salmonelles ont été repiquées sur gélose Cœur-Cervelle pour confirmation par des tests d´identification [22].

**Identification des bactéries**: les colonies bactériennes jaunes sur gélose Hektoen ont été soumises à l´observation microscopique (état frais et coloration de Gram), aux tests enzymatiques (oxydase, catalase, nitrate-réductase) et sur Kligler Iron Agar (dégradation du lactose, fermentation du glucose en anaérobiose avec ou sans production de gaz, production ou non de sulfure d´hydrogène) pour confirmer leur appartenance au groupe des coliformes. Les isolats sur gélose Cœur-Cervelle ont été identifiés à l´aide des tests biochimiques de la galerie Api 20E (BioMérieux, France) pour confirmer les colonies suspectes de salmonelles et *Escherichia coli*. La galerie API 20E est un système standardisé de 20 tests biochimiques. Le principe est la mise en évidence des activités métaboliques des bactéries par des tests biochimiques. Ces tests sont utilisés en laboratoire pour l´identification des *Enterobacteriaceae* et autres bacilles à Gram négatif. A partir d´une culture jeune de 24h sur gélose Cœur-Cervelle, une suspension bactérienne dense a été préparée dans 5ml d´eau physiologique stérile. Les microtubes de la galerie Api 20E ont été ensuite remplis par cette suspension à l´aide d´une micropipette, en évitant la formation de bulles d´air qui empêcheraient le contact entre les bactéries à identifier et le réactif ou substrat à tester. La galerie ensemencée a été incubée à 37^°^C pendant 24h.A l´issue de l´incubation, les résultats de toutes les réactions ont été notés. L´identification bactérienne a été réalisée à l´aide du catalogue analytique du fabricant.

**Analyse des données**: l´analyse des correspondances multiples et la classification hiérarchique sur composantes principales ont été faites sur les données de l´enquête à l´aide du package Rcmdr au moyen du logiciel R 3.4.1 (Rcore Team). Ceci dans le but de déterminer les facteurs qui caractérisent chacun des quartiers, de voir le degré de similarité entre les quartiers et de les classer en groupes ou strates afin de choisir des sites de capture de mouches représentatifs de la zone d´étude. Le test de corrélation de Spearman a été effectué pour évaluer la relation entre les abondances des espèces de mouches. Le test d´ANOVA (analyse de la variance) à plusieurs facteurs a été effectué pour comparer les moyennes des abondances d´espèces de mouches et des bactéries portées par ces mouches selon les lieux, la période et la saison de capture. L´effort d´échantillonnage, les indices de Shannon, de Simpson et de Piélou et Sorensen ont été calculés sur les données des abondances de mouches à l´aide du logiciel ESTIMATE.

## Résultats

**Figure 1:**
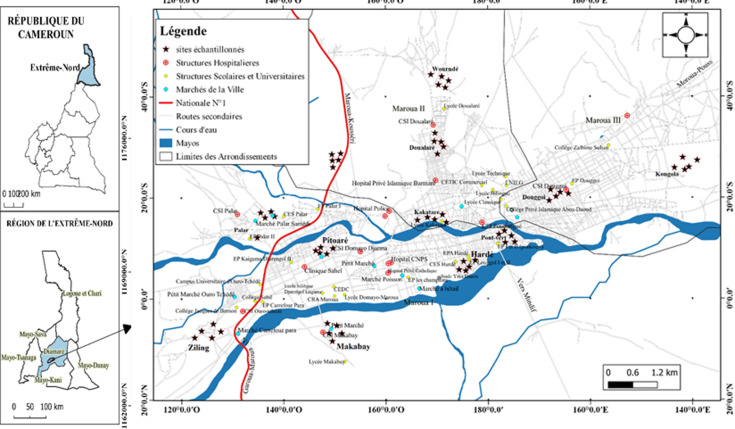
localisation des sites et points de capture des mouches sur la carte de la ville de Maroua

**Sites de capture des mouches**: l´analyse des correspondances multiples (ACM) et la classification hiérarchique sur composantes principales (CHCP) ont permis de regrouper tous les quartiers de la ville en sept strates en fonction des données de l´enquête. Il en ressort que la source majeure de pollution, le type d´assainissement, le type d´ouvrage d´assainissement majoritaire, les défécations humaines à l´air libre aux alentours des habitations, la forte densité de la population sont des paramètres clés dans la différentiation des quartiers. Les facteurs climatiques locaux notamment la température, l´humidité et les précipitations ont été aussi déterminants. La CHCP a permis d´obtenir 7 groupes de quartiers homogènes. Deux quartiers (représentatifs) ont été choisis pour cette étude chaque fois que cela était possible tout en prenant le soin de sélectionner des quartiers éloignés les uns des autres afin de couvrir toute la zone d´étude. Les sites de capture des mouches dans les quartiers retenus sont présentés à la Figure 1.

**Figure 2:**
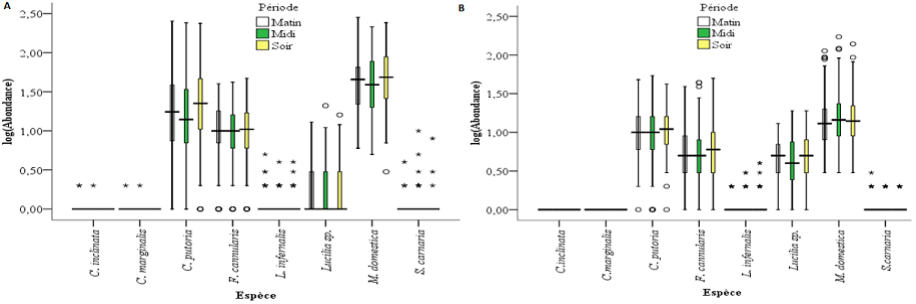
variation des abondances des espèces de mouches en fonction des périodes de la journée et des saisons de l’année

**Figure 3:**
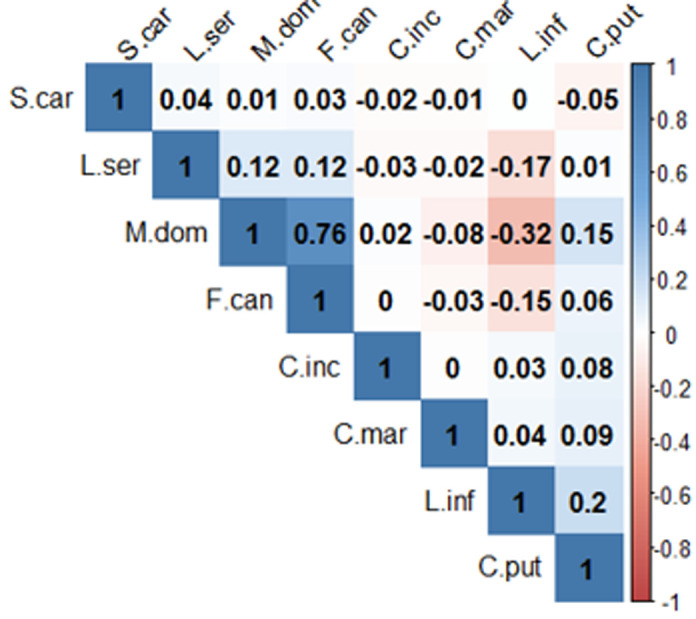
coefficients de corrélation de Spearman (r) entre les abondances des différentes espèces de mouches; (S. car= S. carnaria, L. ser=Lucilia serricata, M. dom=M. domestica, F. canicularis, C. inc= C. inclinata, C. mar= C. marginalis, L. inf= L. infernalis, C. put= C. putoria)

**Structure et biodiversité des communautés des mouches synanthropes**: au cours de cette étude, huit (08) espèces de mouches synanthropes ont été recensées: *Fannia canicularis; Musca domestica; Lucilia sp.; Chrysomya putoria; Lucilia infernalis; Sarcophaga carnaria; Chrysomya marginalis et Chrysomya inclinata*. La courbe d´accumulation a atteint son plateau asymptotique, preuve que le nombre d´espèces de mouches attirées par le substrat utilisé a été atteint. Les abondances de chaque espèce des mouches sont présentées à la Figure 2. *M. domestica* et *F. canicularis* ont été plus abondantes à Hardé, Pont-vert et Doualaré; *C. putoria* a été plus abondante à Kongola, Kakataré et à Makabaye. Les indices de Simpson (D), Shannon (H´) et de Piélou (J) ont présenté respectivement les valeurs de 0,61; 1,10 et 0,53. Les populations des mouches ont montré une saisonnalité relative. L´abondance des mouches à la saison pluvieuse est de 37 250 soit 70,9 % et celle de la saison sèche de 15 255 soit 29,1 %. La différence entre les richesses spécifiques des deux saisons est significative (P<0,05). Trois espèces ont été les plus abondantes durant les deux saisons, dans l´ordre décroissant *M. domestica, C. putoria* et *F. canicularis* (Figure 2). L´indice de diversité de Simpson montre que les populations de *F. canicularis, M. domestica, C. putoria* et de *Lucilia sp*. ont été les plus stables avec les valeurs de 6,19; 6,11; 5,94 et 5,92 respectivement. *C. marginalis* et *C. inclinata* ont été capturées en faible nombre en saison de pluies, respectivement 02 et 03 individus, et ont été totalement absentes en saison sèche. Six espèces ont été enregistrées dans la soirée, huit dans la matinée, et à mi-journée. 36,65% des mouches ont été échantillonnées dans la soirée, 31,1% à mi-journée et 32,16% dans la matinée. *M. domestica, C. putoria* et *F. canicularis* ont été les espèces les plus abondantes à toutes les tranches horaires (Figure 2). *M. domestica* était la plus dominante à la mi-journée avec un pourcentage de 55,11%. Les valeurs des indices de Shannon et de Simpson ont été plus élevées en matinée : H´ = 1,12 et D = 0,62 (avec J = 0,54) alors que celle de l´indice de Piélou a été plus importante en soirée: J = 0,61 (avec H´ = 1,10 et D = 0,60). La Figure 3 montre qu´il y a une affinité entre les espèces de mouches deux-à-deux. Les espèces *M. domestica/F. canicularis; M. domestica/L. serricata ; M. domestica/C. putoria; L. infernalis/C. putoria et L. serricata/F. canicularis* sont retrouvées ensemble, leurs distributions et leurs abondances sont positivement corrélées (P<0,05). Alors que les couples *L. infernalis/M. domestica; L. infernalis/F. canicularis; L. infernalis/L. serricata* sont corrélés négativement (P<0,05).

**Figure 4:**
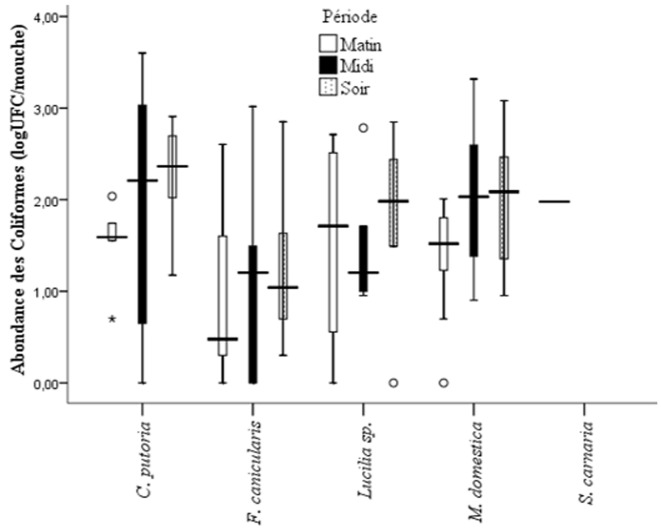
variation des abondances des coliformes portés par espèce de mouche et par période de la journée

**Figure 5:**
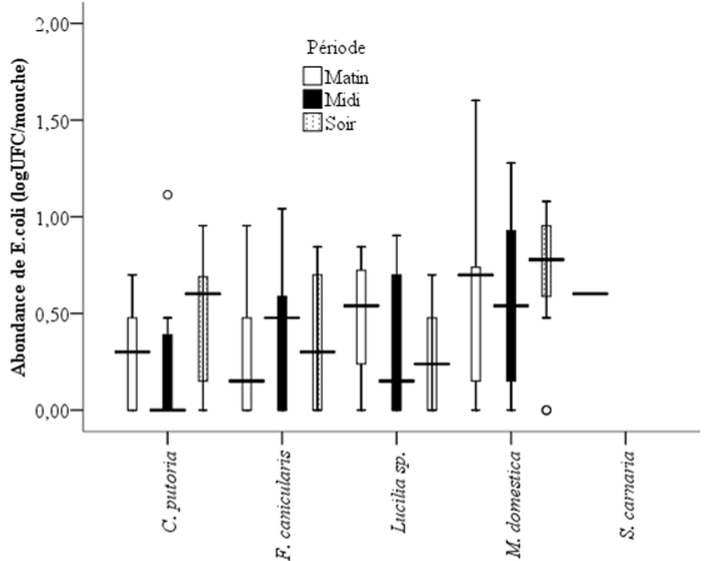
variation des abondances de *Escherichia coli* porté par espèce de mouche et par période de la journée

**Potentiel de transmission des maladies diarrhéiques par les mouches synanthropes et influences des facteurs environnementaux locaux**: les bactéries coliformes et deux entérobactéries dont *Escherichia coli* (indicatrice d´une contamination fécale) et *Salmonella* (bactérie pathogène) ont été recherchées, identifiées et dénombrées. Les analyses de laboratoire ont montré que les mouches étudiées ont toutes été contaminées par des coliformes (Figure 4). Il y a eu une différence significative entre les abondances des coliformes en fonction des quartiers (P<0,05). Les coliformes ont été plus retrouvés sur les mouches de Douggoi, de Doualaré et de Wourndé avec les valeurs respectives de 5156, 4090 et 4061 UFC/mouche, et très faibles à Ziling (250 UFC/mouche). *M. domestica, C. putoria, F. canicularis et Lucilia sp*. sont dans l´ordre décroissant les espèces de mouches synanthropes véhiculant plus de coliformes notamment dans les quartiers Douggoi; Wourndé, Pitoaré, Kongola et Doualaré (Figure 4). Par contre, dans les quartiers Makabaye, Ziling et Frolina, ces mouches véhiculent très faiblement les coliformes. La période la plus favorable au transport des coliformes par les mouches a été la matinée dans les quartiers Wourndé, Douggoi, Kongola et Doualaré; alors que dans les quartiers tels que Pitoaré, Palar et Pont-vert la soirée a été la période de la journée où les coliformes ont été plus abondants sur les mouches. Les quartiers dont les mouches ont présenté les abondances en *Escherichia coli* les plus élevées étaient Douggoi, Pont-vert et Pitoaré avec respectivement 46; 41 et 40 UFC/mouche. *M. domestica* était principalement la plus apte à porter *Escherichia coli* à Pitoaré, Kongola, Pont-vert et Frolina; *C. putoria* à Wourndé et au Pont-vert; *Lucilia sp*. à Pitoaré; *F. canicularis* à Kongola et *S. carnaria* à Makabaye (Figure 5). Le portage de *Escherichia coli* par les mouches a été fonction de la période de la journée. Il y a eu plus de *Escherichia coli* sur les mouches le matin à Makabaye et Pitoaré ; à la mi-journée à Pitoaré et au Pont-vert et à Wourndé et à Kongola que dans la soirée. Les individus de l´espèce *M. domestica* ont été les plus aptes à porter *Escherichia coli* à la mi-journée qu´en soirée. Il existe une différence statistiquement significative entre les abondances moyennes de *Escherichia coli* sur *M. domestica* et sur les autres espèces des mouches (P<0,05). Comme dans le cas des coliformes, les abondances de *Escherichia coli* sur les mouches ont été plus élevées à la mi-journée et en soirée que dans la matinée (P<0,05). Au total, 42 isolats de *Salmonella* ont été collectés sur toutes les espèces de mouches répertoriées. Les salmonelles ont été plus abondantes dans la matinée avec 48 % de l´effectif total. *Lucilia sp*. a été la plus souillée de toutes les espèces. Elle a porté 50 % des salmonelles dénombrées; elle a été suivie de l´espèce *M. domestica* qui a porté 44 % des salmonelles identifiées. Le portage des salmonelles a été plus important dans la matinée (à Pitoaré et à Douggoi), mais beaucoup plus à la mi-journée à Kongola, Douggoi, Kakataré et Hardé.

## Discussion

**Diversité biologique des mouches synanthropes et influences des facteurs environnementaux locaux**: quelques-unes des espèces de mouches synanthropes trouvées ont déjà été identifiées par de nombreux auteurs dans les études antérieures au Cameroun. Le genre *Fannia* a été identifié au Cameroun par les études de Disney [23]. Le genre *Sarcophaga* et les espèces *L. infernalis* et *C. inclinata* ont été identifiés au sud du Cameroun [24]. Toutes ces études n´ont cependant pas été faites dans le but d´estimer la diversité biologique de ces mouches. La valeur de l´indice de dominance (D>0,5) trouvée au cours de cette étude montre qu´au moins une espèce de mouche était plus abondante sur toutes les deux saisons. Cette espèce est *M. domestica* car elle a une abondance élevée (53,5 %) suivie de *C. putoria* avec une abondance de 29,66 %. Ce qui est en accord avec les études menées par Chaiwong *et al*. [25] en Thaïlande et Bawa *et al*. [8] au Togo où *M. domestica*, l´espèce phare était aussi la plus abondante. La dominance de *M. domestica* serait liée à son degré élevé d´affinité aux milieux urbains et péri-urbains que les autres espèces synanthropes, à la disponibilité de ses aliments issus des activités anthropiques. Toutefois, le substrat utilisé peut manquer d´attirer certaines espèces et cela pourrait masquer leur présence dans l´environnement [26].

Les abondances élevées des mouches synanthropes en saison pluvieuse montreraient qu´elles ont plus de chance de survie, notamment avec une bonne température associée à une bonne humidité, la présence de matière organique en décomposition servant de sources d´alimentation et de gites larvaires. Ces résultats sont en accord avec ceux de Keiding [27] et de Richards *et al*. [28] pour qui la présence des fèces humaines, des cadavres d´animaux en état de décomposition associée à la bonne température et à la bonne humidité au début de la saison pluvieuse favoriserait la multiplication de ces mouches. La présence de certaines espèces de mouches dans les deux saisons s´expliquerait par l´existence de nombreux sites d´approvisionnement alimentaire et de pontes ; la tolérance d´un large intervalle de température qui, attribueraient à ces espèces une plasticité écologique importante comme le montrent les études de Archer et Elgar [29]. Selon Williams et Villet [26], l´absence totale de *C. marginalis* et *C. inclinata* en saison sèche pourrait être liée à l´absence des précipitations, au faible degré d´humidité et à la raréfaction de la matière organique en décomposition (séchée par le soleil). Une variation des espèces et d´abondances des mouches en fonction des quartiers de Maroua a été observée au cours de cette étude. *M. domestica* et *F. caniculari s*ont été plus abondantes à Hardé, Pont-vert et Doualaré; *C. putoria* a été plus abondante à Kongola, Kakataré et à Makabaye. Hardé est un quartier traversé par un effluent industriel, et Kongola, quartier d´élevage de bovins; les intenses activités de transformation agroalimentaire (vente du bil-bil, une bière artisanale locale, la présence de nombreux sites de vente de viande fraiche et braisée) au Pont-vert et à Doualaré (contrairement à Frolina) seraient les principaux facteurs favorables à la prolifération d´espèces de mouches dans lesdits quartiers [17,30]. Les abondances de *M. domestica*, et de *C. putoria* renseigneraient que le biotope serait parsemé par des défécations humaines et animales à l´air libre et que les latrines seraient mal entretenues [31-32].

D´après cette étude, les mouches synanthropes ont été plus abondantes dans la soirée. Ce résultat est contraire à celui de Bawa *et al*. [8] qui ont trouvé que les mouches synanthropes étaient plus abondantes en matinée dans les marchés alimentaires et en forêt. La photopériode pourrait déclencher la diapause des mouches pendant la période hivernale et les entrainer en hibernation [33]. De la même façon, le niveau d´ensoleillement pourrait ralentir, activer ou accélérer les activités des mouches, ce qui expliquerait les différences entre les abondances entre les trois périodes de captures de la journée. Les associations entre les espèces de mouches pourraient s´expliquer par leur co-attachement à au moins une source alimentaire et/ou à un facteur qui les obligerait à se tolérer [32]. Ceci pourrait être lié à la différence de régimes alimentaires et à la préférence particulière d´une gamme de température et d´humidité. *C. inclinata* est très faiblement associée à *C. marginalis*. De plus la présence de ces deux espèces a été signalée uniquement en saison pluvieuse. Selon Jackson *et al*. [34], il s´agirait des espèces du climat équatorial beaucoup plus en Amérique latine. Leur présence dans la ville de Maroua montrerait que ces espèces sont en train d´envahir le climat tropical d´où la présence de plusieurs espèces de Calliphoridés et *S. carnaria*, espèce hautement nécrophage. Le niveau de décomposition de l´appât aurait aussi influencé l´abondance de *S. carnaria*. La présence de *F. canicularis* pourrait être associée à des zones de carcasses animales et des végétaux en décomposition, potentiels gîtes larvaires. Les chevauchements observés entre les habitats dans cette étude peuvent être indicateurs de la plasticité environnementale des espèces de mouches synanthropes en raison de leur forte capacité de vol et au degré de synanthropisation [31].

**Potentiel de transmission des maladies diarrhéiques par les mouches synanthropes et influences des facteurs environnementaux locaux**: toutes les mouches ont porté des coliformes. La présence des bactéries du groupe des coliformes dans les sites échantillonnés témoigne d´une contamination par la matière organique (animale et/ou végétale) [35]. La présence d´un taux de portage élevé de coliformes par ces mouches dans les quartiers Kongola, Doualaré, Pitoaré et Douggoi s´expliquerait par l´existence d´importantes activités de transformations agroalimentaires et par la pratique des activités agricoles. Ces résultats sont en accord avec ceux de Craun *et al*. [35] qui ont montré que l´environnement physique urbain et péri-urbain est le plus contaminé par des coliformes. Leurs abondances en matinée et dans la soirée correspondraient aux heures de défécation des bovins (à Kongola et à Wourndé) et aux périodes d´activités agroalimentaires comme la vente des boissons traditionnelles (à Pitoaré, Palar et au Pont-vert), potentielles sources des coliformes [25,36]. Le fait que les abondances des coliformes soient plus élevées pour les espèces de mouches les plus capturées révèlerait que le portage des coliformes est densité-dépendant chez les mouches synanthropes. Les quartiers où les abondances de *Escherichia coli* ont été les plus élevées sont caractérisés par la défécation à l´air libre et/ou une mauvaise gestion des ouvrages d´assainissement. Les abondances de *Escherichia coli* ont été comme les résultats de l´enquête préliminaire les avaient prédites. Pitoaré a été le quartier où la défécation humaine à l´air libre au bord du Mayo-Kaliao est la plus importante; Kongola et Makabaye quant-à eux, les quartiers d´élevage des bovins et des porcins, les abondances des *Escherichia coli* y ont été très importantes. Ces résultats corroborent ceux de Mitsuhiro *et al*. [5]. Les salmonelles retrouvées sur les mouches pourraient être issues des petites fermes avicoles et porcines des quartiers Pitoaré, Makabaye et Kongola ou des excréments des animaux après abattage dans les petits marchés alimentaires. Ce qui est en accord avec les études de Abdus *et al*. [37]. Ceci expliquerait aussi le fait que les salmonelles soient beaucoup plus retrouvées en matinée et à la mi-journée, respectivement les périodes d´abattage et d´exposition de la viande à la vente par les bouchers.

**Limites**: le type d´appât utilisé dans cette étude n´aurait pas pu attirer toutes les espèces de mouches synanthropes de la même façon. Il serait intéressant d´envisager l´utilisation de plusieurs types d´appâts pour des travaux ultérieurs. De plus, les virus entériques et les parasites tels que les amibes étant aussi responsables de maladies diarrhéiques, leur portage, par ces mouches, mérite d´être exploré.

## Conclusion

Au cours de cette étude, 8 espèces de mouches synanthropes dont *M. domestica, F. canicularis, S. carnaria, Lucilia sp., L. infernalis, C. putoria, C. marginalis* et *C. inclinata* ont été identifiées dans la ville de Maroua. La distribution de ces espèces et leurs abondances ont été influencées par les saisons, l´état sanitaire des sites et la période de la journée. Huit espèces ont été retrouvées en saison de pluies contre 6 en saison sèche et les plus abondantes sont *M. domestica ; C. putoria* et *F. canicularis*. Les mouches ont été plus abondantes en saison de pluies (70,9%) qu´en saison sèche (29,1%), et ont été plus importantes dans les quartiers où les activités de transformation agroalimentaire sont intenses notamment Pont-vert, Doualaré et Hardé. La soirée a été la période de la journée où les mouches synanthropes ont été plus capturées. *M. domestica* et *C. putoria* en plus d´être les plus abondantes, ont aussi été les plus aptes à porter les microorganismes. Le portage des bactéries fécales par les mouches synanthropes semble donc être abondance-dépendant. La mauvaise gestion des ordures ménagères, les activités de transformations agro-économiques et un mauvais assainissement sont les sources probables de contamination des mouches par les bactéries fécales. Les salmonelles isolées des mouches étant des bactéries pathogènes entériques, il est très probable que les mouches synanthropes soient impliquées dans la dissémination des maladies diarrhéiques à travers la contamination des aliments dans la ville de Maroua.

### Etat des connaissances sur le sujet

- Les mouches synanthropes sont fréquemment incriminées dans la transmission des germes pathogènes à l´Homme à travers la contamination des aliments de ce dernier;
- Ces mouches se nourrissent de la matière organique en décomposition, contenant de nombreuses espèces de microorganismes souvent pathogènes pour l´Homme;
- Les épidémies de maladies diarrhéiques sont fréquentes dans les régions septentrionales du Cameroun.

### Contribution de notre étude à la connaissance

- Contribue à la connaissance de la biodiversité des mouches synanthropes de la ville de Maroua;
- Démontre que les mouches synanthropes transportent en plus des coliformes et Escherichia coli, bactéries indicatrices de contamination fécale des microorganismes pathogènes tels que les salmonelles susceptibles de causer des maladies diarrhéiques à l´homme;
- Permet de savoir que le portage des bactéries par les mouches synanthropes est abondance-dépendant dans la ville de Maroua.

## Remerciements

Les auteurs remercient l´équipe de recherche du Pr Kekeunou Sévilor du laboratoire de Zoologie Appliquée et Approfondie de l´Université de Yaoundé 1 pour sa contribution à l´identification des spécimens des mouches.
